# Administration of antibiotics contributes to cholestasis in pediatric patients with intestinal failure via the alteration of FXR signaling

**DOI:** 10.1038/s12276-018-0181-3

**Published:** 2018-11-30

**Authors:** Yongtao Xiao, Kejun Zhou, Ying Lu, Weihui Yan, Wei Cai, Ying Wang

**Affiliations:** 1 Department of Pediatric Surgery, Xin Hua Hospital, School of Medicine, Shanghai Jiao Tong University, Shanghai, China; 2 Shanghai Institute of Pediatric Research, Shanghai, China; 3 Shanghai Key Laboratory of Pediatric Gastroenterology and Nutrition, Shanghai, China

**Keywords:** Experimental models of disease, Colitis

## Abstract

The link between antibiotic treatment and IF-associated liver disease (IFALD) is unclear. Here, we study the effect of antibiotic treatment on bile acid (BA) metabolism and investigate the involved mechanisms. The results showed that pediatric IF patients with cholestasis had a significantly lower abundance of BA-biotransforming bacteria than patients without cholestasis. In addition, the BA composition was altered in the serum, feces, and liver of pediatric IF patients with cholestasis, as reflected by the increased proportion of primary BAs. In the ileum, farnesoid X receptor (FXR) expression was reduced in patients with cholestasis. Correspondingly, the serum FGF19 levels decreased significantly in patients with cholestasis. In the liver, the expression of the rate-limiting enzyme in bile salt synthesis, cytochrome P450 7a1 (CYP7A1), increased noticeably in IF patients with cholestasis. In mice, we showed that oral antibiotics (gentamicin, GM or vancomycin, VCM) reduced colonic microbial diversity, with a decrease in both Gram-negative bacteria (GM affected *Eubacterium* and *Bacteroides*) and Gram-positive bacteria (VCM affected *Clostridium*, *Bifidobacterium* and *Lactobacillus*). Concomitantly, treatment with GM or VCM decreased secondary BAs in the colonic contents, with a simultaneous increase in primary BAs in plasma. Moreover, the changes in the colonic BA profile especially that of tauro-beta-muricholic acid (TβMCA), were predominantly associated with the inhibition of the FXR and further altered BA synthesis and transport. In conclusion, the administration of antibiotics significantly decreased the intestinal microbiota diversity and subsequently altered the BA composition. The alterations in BA composition contributed to cholestasis in IF patients by regulating FXR signaling.

## Liver disease: Detrimental effects of antibiotics in intestinal failure

Using antibiotics during intestinal failure in children may lead to the development of liver disease. Microbiota in the gut play vital roles in balancing the digestive system, including transforming bile acids (BAs) secreted by the liver into forms that help us digest food. Wai Cai and Ying Wang at Shanghai Jiao Tong University in China and co-workers examined samples from 46 children treated with antibiotics for intestinal failure. The patients who also had cholestasis – disrupted production and flow of bile – had far fewer BA-transforming bacteria in their gut than those without cholestasis. They also had altered expression of a crucial BA receptor protein. Experiments on mice showed that treatment with two different antibiotics reduced microbiota diversity, which in turn influenced BA receptor signaling and altered BA composition, contributing to cholestasis.

## Introduction

Intestinal failure (IF)-associated liver disease (IFALD) is a serious complication in pediatric patients with IF^1,2^. We and others previously showed that cholestasis could cause liver injury in pediatric IF patients^3–5^. However, the mechanisms underlying the development of cholestasis are poorly understood. In clinics, antibiotics are widely used to prevent bacteremia in pediatric IF patients^6^. Antibiotic treatment may lead to intestinal dysbiosis. To unravel the links between cholestasis and antibiotic administration, we performed a population-based cross-sectional study in pediatric IF patients treated with antibiotics.

The human intestinal microbiota, which is essential to maintain host energy metabolism and immune functions, consists of over 1000 bacterial species^7,8^. Prebiotics, probiotics, and antibiotics can modulate the gut microbiota composition^9^, thereby altering the presence and expression of microbial genes and derived metabolites^10^. Particularly, the use of antibiotics has been associated with increased metabolic impairments, mainly when exposure occurs in early life^11^. Oral antibiotics can result in short-term and long-term changes in the intestinal microbiota in both humans and mice^12,13^. It has recently been reported that antibiotics may improve peripheral insulin sensitivity in a small number of obese subjects^14^. Correspondingly, by altering the gut microbiota, antibiotics also induce profound changes in bile acid (BA) metabolism^14,15^. Bile acids are synthesized by hepatic enzymes from cholesterol and are important for lipoprotein, glucose, drug, and energy metabolism^16^. Mice synthesize two primary BAs, cholate (CA) and muricholate (MCA), whereas humans synthesize CA and chenodeoxycholate (CDCA). Primary BAs are further conjugated with taurine and glycine^17^. Once made in the liver, 95% of primary BAs, unconjugated and conjugated, are absorbed in the terminal ileum and returned to the liver^16^. Primary BAs that reach the large intestine are biotransformed by members of the gut microbiota via two enzymatic reactions, deconjugation and dehydroxylation, into secondary BAs, including MCA, hyodeoxycholate (HDCA), ursodeoxycholate, (UDCA), lithocholate (LCA), and deoxycholate (DCA)^18^.

To date, it is poorly understood whether and to what extent intestinal bacteria are involved in the regulation of human BA homeostasis. In view of the different roles of Gram-positive and Gram-negative bacteria in intestinal BA metabolism, the modification of either of these bacteria may have distinct effects on BA homeostasis. The farnesoid X receptor (FXR, also known as NR1H4) is known to play a key role in the regulation of BA synthesis and homeostasis^19^. We thus hypothesize that the intestinal microbiota composition can affect the BA composition with consequent alterations in FXR signaling, thereby affecting BA metabolism and leading to cholestasis in IF patients. In the present study, we evaluated the impact of two different antibiotic regimens known to protect against Gram-positive and Gram-negative bacteria (vancomycin, VCM and gentamicin, GM; orally administered) on the intestinal microbiota composition and BA metabolism. We showed that the reduction in both Gram-positive and Gram-negative bacteria by VCM and GM was associated with BA dysmetabolism and that the modulation of FXR signaling may be instrumental in mediating this effect.

## Materials and methods

### Patients

A total of 46 pediatric patients with IF were enrolled in this study. Serum samples and tissues were obtained from patients who underwent surgery. All patients’ guardians provided written informed consent. This study was approved by the Faculty of Medicine’s Ethics Committee of Xin Hua Hospital, School of Medicine, Shanghai Jiao Tong University, Shanghai, China. All methods in this study were carried out in accordance with the relevant guidelines.

### Antibiotic treatment

Six-week-old C57BLl/6 mice were treated with gentamicin (2 g/L; Nacalai Tesque, Kyoto, Japan) or vancomycin (500 mg/L; Duchefa Biochemie B.V.) dissolved in autoclaved drinking water and provided for 2 weeks. The fluid intake and body weight were monitored. All procedures were approved by the Shanghai Jiao Tong University School of Medicine affiliated Xin Hua Hospital Animal Care and Use Committee.

### Histology and immunohistochemistry (IHC)

Histological examination involved staining with hematoxylin and eosin (H&E). IHC was performed using the diaminobenzidine (DAB) as the chromogen. Briefly, paraffin-embedded tissues were deparaffinized using xylol and descending concentrations of ethanol. Citrate buffer (pH 6.0) was used for antigen retrieval. Endogenous peroxidases were removed by 0.3% H_2_O_2_ and then blocked using 5% bovine serum albumin (BSA). Primary antibodies were applied at an optimal concentration overnight in a wet chamber (Cyp7a1, Millipore, Darmstadt, Germany, dilution, 1:300; FXR, Invitrogen, dilution 1:500). Antibody binding was visualized by a liquid DAB substrate chromogen system (Dako, Glostrup, Denmark).

### Quantitative PCR amplification of 16S rRNA genes

After the weights were measured, bacterial DNA was extracted from colonic content using a QIAamp Fast DNA Mini Kit (Qiagen). Quantitative PCR was performed in an ABIViiA 7 instrument using a SYBR Green Universal Master Mix kit. The following primer sets were used: ‘all bacteria’, 5′-CGGTGAATACGTTCCCGG-3′ and 5′-TACGGCTACCTTGTTACGACTT-3′; *Clostridium*, 5′-GGGAGTACGGTCGCAAGATT-3′ and 5′-ATGCACCACCTGTCTTCCTG-3′; *Eubacterium*, 5′-GGGGAGTACGTTCGC-AAGAA-3′ and 5′-GCTCCGAAGAGAAGGTACGG-3′; *Bifidobacterium*, 5′-CTCCTGGAAACGGGTGG-3′ and 5′-GGTGTTCTTCCCGATATCTACA-3′; *Lactobacillus*, 5′-TGGAAACAGRTGCTAATACCG-3′ and 5′-GTCCATTGTGGAAGATTCCC-3′; *Bacteroides*, 5′-GAGAGGAAGGTCCC-CCAC-3′ and 5′ -CGCTACTTGGCTGGTTCAG-3′.

### Western blotting

Equal amounts of proteins were separated by 10% SDS-PAGE and transferred to polyvinylidene difluoride (PVDF) membranes. After blocking in 5% nonfat dry milk, 0.2% Tween 20 at room temperature (RT) for 30 min, membranes were incubated overnight at 4 °C with primary antibodies. Antibodies against Cyp7a1 (dilution, 1:100), Fxr, (dilution, 1:200) and β-actin (dilution, 1:500) were analyzed. The membranes were washed with PBS (containing 0.1% Tween) and incubated with horseradish peroxidase-conjugated secondary antibody. The antigen-antibody complexes were detected using an ECL Plus chemiluminescence reagent kit (Pierce, Rockford, IL, USA).

### Statistical analysis

The statistics are presented as the medians with IQRs or as the means ± SDs. The Kolmogorov–Smirnov test was used to assess distributions. The Mann–Whitney *U* test, Fisher’s exact test or one-way ANOVA were used to compare differences between groups. The level of statistical significance was set at 0.05.

Additional protocols are presented in the Supplementary Materials and Methods.

## Results

### Bile acid-biotransforming bacteria are decreased in IF patients with cholestasis

**Fig. 1:**
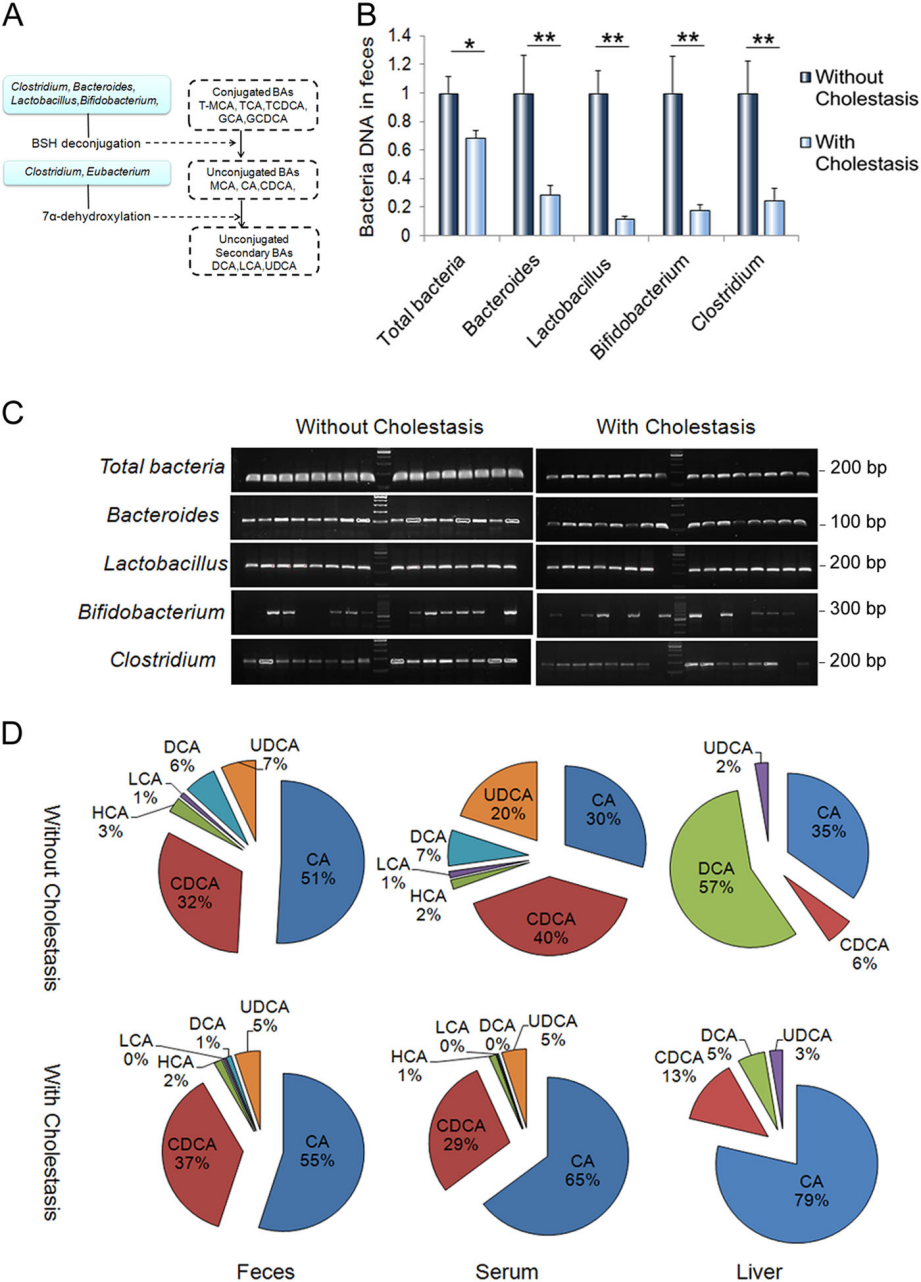
Intestinal failure (IF) patients with cholestasis exhibit alterations in bile acid (BA)-biotransforming bacteria and BA composition. **a** Schematic depicting the roles of intestinal bacteria in BA metabolism. **b**, **c** The abundances of BA-biotransforming bacteria in the feces of patients with cholestasis (n = 16) were significantly lower than those in the feces of subjects without cholestasis (*n* = 16). **d** Changes in the BA composition in serum (without cholestasis, *n* = 26, with cholestasis, *n* = 20), feces (without cholestasis, *n* = 16, with cholestasis, *n* = 16) and liver tissue (without cholestasis, *n* = 12, with cholestasis, *n* = 8). *p < 0.05, **p < 0.01

As shown in Table 1, abnormal values of liver enzymes and parameters of cholestasis were observed in IF patients (Table 1). The values of liver enzymes, including ALP (240.5 (201.5–286.5) U/L vs. 275 (173–311.6) U/L), ALT 45 (31.5–89) U/L vs. 56 (33.6–97) U/L, *p* < 0.01) and AST 59 (47.9–98.7) U/L vs. 67 (56.5–114.5) U/L, *p* < 0.01) were higher in patients with cholestasis than in patients without cholestasis (Table 1). Plasma total bilirubin (9.7 (9.2–18.5) μmol/L vs. 13.5 (9.5–34.2) μmol/L, *p* = 0.01) and conjugated bilirubin (2.3 (0.2–5.2) μmol/L vs. 10.5 (9.3–22.5) μmol/L, *p* = 0.03) were also upregulated in patients with cholestasis (Table 1). In addition, the proinflammatory factors serum interleukin-6 (IL-6) (4.2 (4.6–7.8) pg/ml vs. 7.7 (4.3–41.5) pg/ml, *p* < 0.01) and tumor necrosis factor-alpha (TNF-α) (6.9 (0.7–14.7) pg/ml vs. 8.6 (7.3–15.4) pg/ml, *p* < 0.01) concentrations were higher in patients with cholestasis than in those without cholestasis (Table 1). Interestingly, we noticed that patients with cholestasis had longer durations of antibiotic administration (4 (6–16) vs. 4 (3–9) days, *p* < 0.01) than the patients without cholestasis. In accordance with the prolonged antibiotic administration, the intestinal bacteria were altered significantly in patients with cholestasis (Fig. 1a–c). The main bacterial genera of the gut microbiota involved in BA metabolism include *Bacteroides* (Gram-negative), *Clostridium* (Gram-positive), *Lactobacillus* (Gram-positive), *Bifidobacterium* (Gram-positive) and *Eubacterium* (Gram-negative). The bacteria *Bacteroides*, *Clostridium*, *Lactobacillus*, and *Bifidobacterium* deconjugate taurine-conjugated and glycine-conjugated BAs to their respective unconjugated free forms through the action of bile salt hydrolase (BSH)^16^. *Clostridium* and *Eubacterium* convert unconjugated primary bile acids into secondary bile acids through 7α‑dehydroxylation (Fig. 1a)^20^. The abundances of *Bacteroides, Lactobacillus, Bifidobacterium*, and *Clostridium* were all significantly lower in the feces of patients with cholestasis than in the feces of those without cholestasis (Fig. 1b, c).

**Table 1:** Characteristics of the patients

Variable	Patients without cholestasis	Patients with cholestasis	*p* value^a^
Patients (*n*)	26	20	0.25
Short bowel syndrome (*n*)	6	9	0.59
Male (*n*)	16	11	0.69
Age (months)	5 (3.3–6)	5 (3.1–7)	0.87
Gestational age (weeks)	37 (31.5–38)	36 (30–39)	0.58
Gestational weight (g)	2900 (1486–3162)	2650 (2030–3025)	0.08
Plasma alkaline phosphatase, ALP (U/L)	240.5 (201.5–286.5)	275 (173–311.6)	<0.01
Plasma alanine aminotransferase, ALT (U/L)	45 (31.5–89)	56 (33.6–97)	<0.01
Plasma aspartate aminotransferase, AST (U/L)	59 (47.9–98.7)	67 (56.5–114.5)	<0.01
Plasma total bilirubin (μmol/L)	9.7 (9.2–18.5)	13.5 (9.5–34.2)	0.01
Plasma conjugated bilirubin (μmol/L)	2.3 (0.2–5.2)	10.5 (9.3–22.5)	0.03
Serum IL-6 (pg/mL)	4.2 (4.6–7.8)	7.7 (4.3–41.5)	<0.01
Serum TNF-α (pg/mL)	6.9 (0.7–14.7)	8.6 (7.3–15.4)	<0.01

The data are the medians (ranges)

^a^Comparison between patients with and without cholestasis using Fisher’s exact test or the Mann–Whitney *U* test

### FXR signaling is altered in IF patients with cholestasis

In accordance with the microbial results, the proportion of primary BAs (CA and CDCA) observed in feces of patients with cholestasis was higher than in the subjects without cholestasis (Fig. 1d and Supplementary Table 1). Conversely, the proportion of all secondary/tertiary BAs, including DCA, LCA, and UDCA, decreased in patients with cholestasis (Fig. 1d and Supplementary Table 1). In the serum of patients with cholestasis, the BA profiles shifted to a primary BA-dominant composition, with significant increases in the levels of conjugated and unconjugated CDCA and CA along with significantly decreased levels of unconjugated and conjugated LCA, DCA, and UDCA (Fig. 1d and Supplementary Table 2). In the liver of patients with cholestasis, both unconjugated and conjugated primary BAs, including CA and CDCA, increased noticeably (Fig. 1d and Supplementary Table 3).

**Fig. 2:**
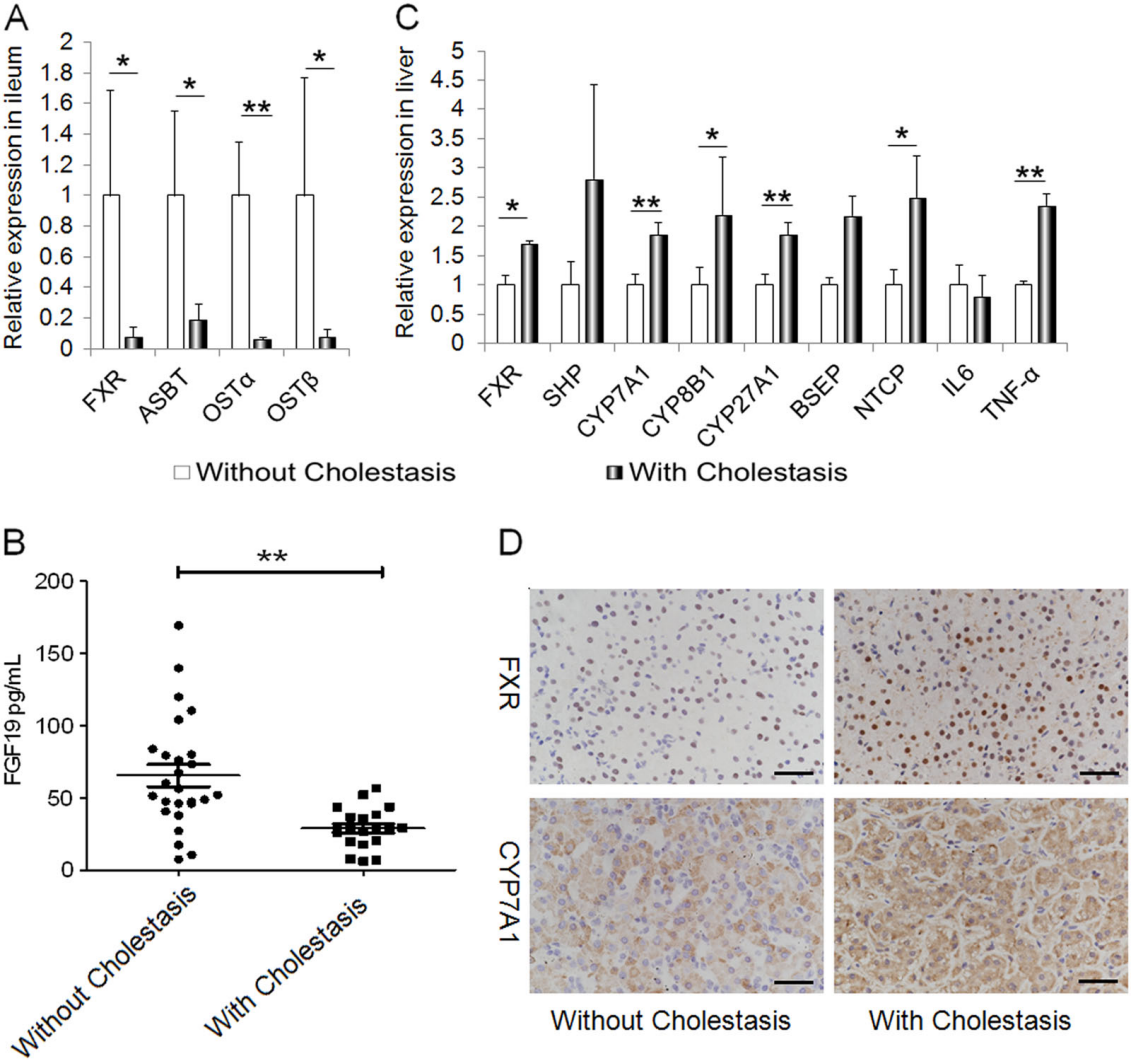
Intestinal failure (IF) patients with cholestasis exhibit disrupted FXR signaling. **a** The expression of FXR and its target genes in the ileum was significantly lower in patients with cholestasis (*n* = 12) than in subjects without cholestasis (*n* = 8). **b** Serum FGF19 decreased in patients with cholestasis (*n* = 26) compared to those without cholestasis (*n* = 20). **c** Alterations in the expression of FXR and downstream genes in liver tissues (without cholestasis, *n* = 16, with cholestasis, *n* = 12). **d** Representative images of FXR and CYP7A1 immunohistochemical staining in liver tissues of patients. Scale bar = 25 µm; *p < 0.05, **p < 0.01

Given that the BA composition is known to strongly influence FXR activation, we further analyzed the changes in FXR signaling in patients. As shown in Fig. 2a, the expression of the FXR- and the FXR-targeted genes ASBT and OSTα/β was significantly lower in the ileum of patients with cholestasis than in the ileum of patients without cholestasis (Fig. 2a). The level of an important FXR target gene, FGF19, which is essential to the intestine-driven repression of hepatic CYP7A1, was reduced significantly in the serum of patients with cholestasis (Fig. 2b). In accordance with the decrease in FGF19 in patients with cholestasis, BA-synthesized enzymes, including CYP7A1, CYP8B1, and CYP27A1, increased significantly in the liver (Fig. 2c, d).

### Antibiotic treatments decreased the abundance of bile acid-biotransforming bacteria in mice

Mice were treated with oral gentamicin (GM, 2 g/L) or vancomycin (VCM, 500 mg/L) for 2 weeks, and their body weights were monitored. As seen in Supplementary Fig 1A, the mice treated with antibiotics, especially those treated with VCM, exhibited slightly higher body weights than the untreated mice (Supplementary Fig 1A). At 2 weeks after antibiotic treatment, both the GM-treated and VCM-treated animals exhibited higher levels of IL-6 and TNF-α than the untreated animals (Supplementary Fig 1B and 1C). The proinflammatory cytokines IL-6 and TNF-α have been shown to be important mediators of cholestatic liver injury^21–23^. Indeed, the mice treated with antibiotics exhibited alterations in liver function and liver histology (Supplementary Fig 1D and 1E).

**Fig. 3:**
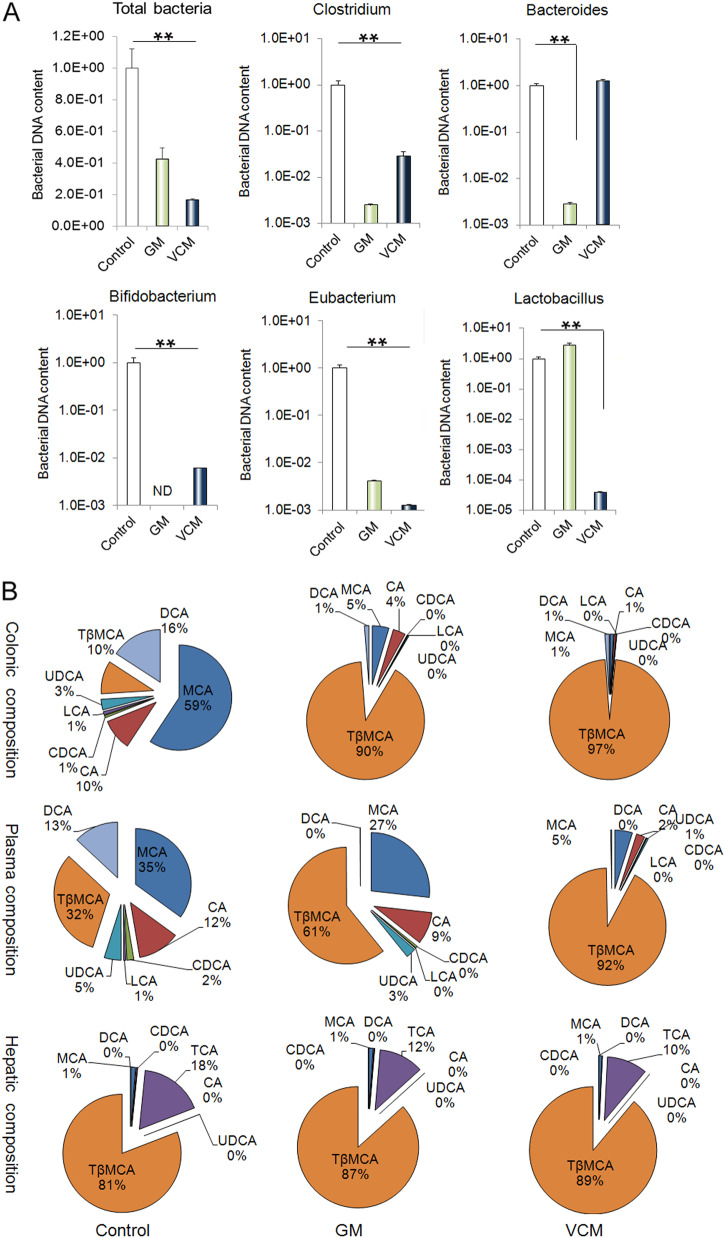
Antibiotic-treated mice exhibit intestinal microbial dysbiosis and bile acid dysmetabolism. **a** Alterations in the relative abundance of bacteria responsible for the biotransformation of bile acids in colonic contents obtained at sacrifice from either untreated or antibiotic-treated animals. **b** Changes in the bile acid composition in colonic contents, serum or liver obtained at sacrifice from untreated and antibiotic-treated animals. **p < 0.01

VCM is an antibiotic that mostly affects Gram-positive bacteria, while GM mostly affects Gram-negative bacteria. After two weeks of oral GM or VCM treatment, the mice exhibited significantly reduced colonic microbial diversity (Fig. 3a). As expected, GM administration decreased the abundance of Gram-negative bacteria (mainly *Eubacterium* and *Bacteroides*). VCM administration reduced the abundance of Gram-positive bacteria (mainly *Clostridium, Bifidobacterium* and *Lactobacillus*) (Fig. 3a). Interestingly, the abundances of *Clostridium, Bifidobacterium* and *Eubacterium* were reduced significantly following GM and VCM administration (Fig. 3a).

### Antibiotic administration alters the bile acid composition in mice

Because alterations in the abundance of BA-biotransforming bacteria were detected in antibiotic-treated mice, we further assessed changes in the BA composition in the colonic content, serum and liver (Fig. 3b). To study whether bacteria-mediated alterations in BA composition affect FXR activation, we assessed the changes in the proportion of BA species with known FXR agonist activity (CDCA, DCA, LCA), those with no agonistic potential (UDCA) and those with antagonist activity (TβMCA) (Tables 2–4).

**Table 2:** Bile acid profiles in the colonic content of mice (nmol/mg)

Bile acid (nmol/mg)	Untreated	GM	VCM	*p* value^a^ (GM vs. untreated)	*p* value^a^ (VCM vs. untreated)
7DHCA	16.49 ± 5.95	49.26 ± 71.74	0.76 ± 1.09	0.191	0.076
12DHCA	2.10 ± 1.23	1.99 ± 3.02	0.89 ± 1.32	0.922	0.357
3DHCA	1.65 ± 0.48	5.12 ± 6.23	0.40 ± 0.56	0.115	0.051
ωMCA	99.09 ± 74.65	19.22 ± 18.57	2.66 ± 5.99	0.007	0.029
α MCA	32.61 ± 15.67	13.99 ± 20.09	6.57 ± 10.65	0.043	0.366
βMCA	103.13 ± 101.17	109.10 ± 120.18	68.23 ± 103.94	0.911	0.468
HCA	4.03 ± 3.83	0.76 ± 0.64	0.24 ± 0.37	0.022	0.064
CA	38.44 ± 40.79	57.36 ± 225.41	46.20 ± 72.06	0.380	0.475
CDCA	2.79 ± 4.63	4.84 ± 11.20	3.99 ± 5.71	0.618	0.849
muroCA	4.20 ± 2.98	0.14 ± 0.17	0.11 ± 0.18	0.001	0.752
Total unconjugated primary BAs	304.53 ± 151.43	261.77 ± 474.62	130.05 ± 198.35	0.966	0.331
TωMCA	9.69 ± 8.50	1.89 ± 1.02	2586.99 ± 4881.97	0.015	0.132
TαMCA	2.76 ± 1.10	1.42 ± 0.90	13.97 ± 16.07	0.012	0.033
TβMCA	40.20 ± 31.75	2812.86 ± 890.14	8187.01 ± 1435.99	0.003	0.007
THCA	0.11 ± 0.08	6.96 ± 20.40	3.73 ± 6.35	0.329	0.674
TCA	4.08 ± 1.75	724.17 ± 2164.71	725.16 ± 1270.96	0.333	0.999
GCDCA	0.03 ± 0.02	0.06 ± 0.12	0.05 ± 0.02	0.567	0.818
GCA	0.05 ± 0.03	0.38 ± 1.10	0.10 ± 0.15	0.379	0.481
TCDCA	3.90 ± 2.25	51.22 ± 121.60	49.13 ± 63.79	0.260	0.966
Total conjugated primary BAs	60.82 ± 32.45	3598.96 ± 1593.35	11566.14 ± 2200.34	0.033	0.031
6ketoLCA	2.76 ± 2.06	0.35 ± 0.14	0.03 ± 0.04	0.003	0.000
7ketoLCA	1.35 ± 0.90	0.74 ± 2.52	0.27 ± 0.38	0.667	0.125
UDCA	12.98 ± 8.66	7.98 ± 13.85	3.94 ± 6.23	0.372	0.459
HDCA	16.78 ± 15.48	0.20 ± 0.19	0.25 ± 0.34	0.005	0.727
DCA	62.74 ± 38.62	0.25 ± 0.28	30.47 ± 116.53	0.000	0.019
Total unconjugated secondary/tertiary BAs	96.61 ± 51.15	10.52 ± 16.50	54.72 ± 46.00	0.000	0.019
TLCA	0.39 ± 0.41	0.05 ± 0.15	0.22 ± 0.44	0.032	0.301
TUDCA	1.27 ± 0.54	0.88 ± 0.59	137.22 ± 250.43	0.164	0.122
THDCA	0.90 ± 0.62	0.07 ± 0.20	0.85 ± 2.19	0.002	0.301
TDCA	6.12 ± 4.07	0.14 ± 0.29	0.43 ± 1.05	0.001	0.449
Total conjugated secondary/tertiary BAs	7.90 ± 5.07	1.14 ± 0.61	138.72 ± 252.73	0.001	0.140

The data are the means ± SDs

^a^One-way ANOVA was used to compare differences between the GM vs. untreated groups and between the VCM vs. untreated groups

**Table 3:** Bile acid profiles in the serum of mice (nmol/L)

Bile acid (nmol/mg)	Untreated	GM	VCM	*p* value^a^ (GM vs. untreated)	*p* value^a^ (VCM vs. untreated)
7DHCA	16.49 ± 5.95	49.26 ± 71.74	0.76 ± 1.09	0.191	0.076
12DHCA	2.10 ± 1.23	1.99 ± 3.02	0.89 ± 1.32	0.922	0.357
3DHCA	1.65 ± 0.48	5.12 ± 6.23	0.40 ± 0.56	0.115	0.051
ωMCA	99.09 ± 74.65	19.22 ± 18.57	2.66 ± 5.99	0.007	0.029
α MCA	32.61 ± 15.67	13.99 ± 20.09	6.57 ± 10.65	0.043	0.366
βMCA	103.13 ± 101.17	109.10 ± 120.18	68.23 ± 103.94	0.911	0.468
HCA	4.03 ± 3.83	0.76 ± 0.64	0.24 ± 0.37	0.022	0.064
CA	38.44 ± 40.79	57.36 ± 225.41	46.20 ± 72.06	0.380	0.475
CDCA	2.79 ± 4.63	4.84 ± 11.20	3.99 ± 5.71	0.618	0.849
muroCA	4.20 ± 2.98	0.14 ± 0.17	0.11 ± 0.18	0.001	0.752
Total unconjugated primary BAs	304.53 ± 151.43	261.77 ± 474.62	130.05 ± 198.35	0.966	0.331
TωMCA	9.69 ± 8.50	1.89 ± 1.02	2586.99 ± 4881.97	0.015	0.132
TαMCA	2.76 ± 1.10	1.42 ± 0.90	13.97 ± 16.07	0.012	0.033
TβMCA	40.20 ± 31.75	2812.86 ± 890.14	8187.01 ± 1435.99	0.003	0.007
THCA	0.11 ± 0.08	6.96 ± 20.40	3.73 ± 6.35	0.329	0.674
TCA	4.08 ± 1.75	724.17 ± 2164.71	725.16 ± 1270.96	0.333	0.999
GCDCA	0.03 ± 0.02	0.06 ± 0.12	0.05 ± 0.02	0.567	0.818
GCA	0.05 ± 0.03	0.38 ± 1.10	0.10 ± 0.15	0.379	0.481
TCDCA	3.90 ± 2.25	51.22 ± 121.60	49.13 ± 63.79	0.260	0.966
Total conjugated primary BAs	60.82 ± 32.45	3598.96 ± 1593.35	11566.14 ± 2200.34	0.033	0.031
6ketoLCA	2.76 ± 2.06	0.35 ± 0.14	0.03 ± 0.04	0.003	0.000
7ketoLCA	1.35 ± 0.90	0.74 ± 2.52	0.27 ± 0.38	0.667	0.125
UDCA	12.98 ± 8.66	7.98 ± 13.85	3.94 ± 6.23	0.372	0.459
HDCA	16.78 ± 15.48	0.20 ± 0.19	0.25 ± 0.34	0.005	0.727
DCA	62.74 ± 38.62	0.25 ± 0.28	30.47±116.53	0.000	0.019
total unconjugated secondary/tertiary BAs	96.61 ± 51.15	10.52 ± 16.50	54.72 ± 46.00	0.000	0.019
TLCA	0.39 ± 0.41	0.05 ± 0.15	0.22 ± 0.44	0.032	0.301
TUDCA	1.27 ± 0.54	0.88 ± 0.59	137.22 ± 250.43	0.164	0.122
THDCA	0.90 ± 0.62	0.07 ± 0.20	0.85 ± 2.19	0.002	0.301
TDCA	6.12 ± 4.07	0.14 ± 0.29	0.43 ± 1.05	0.001	0.449
total conjugated secondary/tertiary BAs	7.90 ± 5.07	1.14 ± 0.61	138.72 ± 252.73	0.001	0.140

The data are the means ± SDs

^a^One-way ANOVA was used to compare differences between the GM vs. untreated groups and between the VCM vs. untreated groups

**Table 4:** Bile acid profiles in the liver of mice (nmol/mg protein)

Bile acid (nmol/mg)	Untreated	GM	VCM	*p* value^a^ (GM vs. untreated)	*p* value^a^ (VCM vs. untreated)
ωMCA	6.53 ± 13.54	10.88 ± 12.30	0.45 ± 0.40	0.501	0.031
αMCA	3.90 ± 6.85	10.80 ± 11.32	1.87 ± 0.92	0.144	0.043
βMCA	19.42 ± 26.35	131.80 ± 151.34	42.01 ± 21.49	0.044	0.119
HCA	0.09 ± 0.20	0.22 ± 0.35	0.04 ± 0.03	0.355	0.160
CA	8.04 ± 18.87	30.30 ± 30.67	1.21 ± 0.42	0.088	0.018
muroCA	0.64 ± 0.70	0.21 ± 0.17	0.04 ± 0.04	0.109	0.019
CDCA	0.16 ± 0.19	0.64 ± 0.74	0.19 ± 0.08	0.078	0.110
7DHCA	7.47 ± 20.52	28.03 ± 47.27	0.12 ± 0.05	0.253	0.117
12DHCA	0.39 ± 0.36	1.32 ± 1.45	0.22 ± 0.16	0.082	0.051
3DHCA	0.03 ± 0.04	0.07 ± 0.10	0.01 ± 0.02	0.338	0.114
Total unconjugated primary BAs	46.68 ± 86.67	214.26 ± 202.73	56.16 ± 22.35	0.039	0.035
TωMCA	134.20 ± 133.85	681.24 ± 1609.90	30.64 ± 35.88	0.324	0.272
TαMCA	201.87 ± 379.71	207.65 ± 561.58	19.96 ± 35.21	0.980	0.361
TβMCA	1950.61 ± 2393.96	11916.86 ± 9826.94	5212.38 ± 3520.19	0.010	0.091
THCA	4.47 ± 8.11	15.14 ± 15.58	1.29 ± 1.34	0.091	0.025
TCA	422.81 ± 460.48	1643.28 ± 1950.49	602.53 ± 445.13	0.088	0.163
GCDCA	0.09 ± 0.10	0.09 ± 0.10	0.04 ± 0.01	0.930	0.202
GCA	0.46 ± 0.65	0.85 ± 0.84	0.08 ± 0.06	0.298	0.022
TCDCA	51.44 ± 53.44	156.11 ± 189.70	59.05 ± 28.44	0.132	0.174
Total conjugated primary BAs	2765.95 ± 3172.88	14621.24 ± 12816.42	5925.97 ± 3934.01	0.017	0.088
6ketoLCA	0.03 ± 0.03	0.06 ± 0.05	0.02 ± 0.02	0.247	0.040
7ketoLCA	0.01 ± 0.01	0.06 ± 0.08	0.01 ± 0.01	0.080	0.071
UDCA	0.60 ± 0.58	3.51 ± 3.50	0.68 ± 0.34	0.026	0.039
HDCA	0.30 ± 0.31	0.15 ± 0.20	0.06 ± 0.03	0.267	0.210
DCA	0.19 ± 0.19	0.13 ± 0.29	0.02 ± 0.03	0.638	0.283
Total unconjugated secondary/tertiary BAs	1.13 ± 1.04	3.91 ± 4.07	0.78 ± 0.41	0.065	0.088
TLCA	4.58 ± 4.03	1.13 ± 0.83	0.62 ± 0.14	0.032	0.112
TUDCA	74.77 ± 83.98	343.98 ± 304.56	110.74 ± 72.67	0.022	0.054
THDCA	28.98 ± 42.55	0.34 ± 0.57	0.27 ± 0.68	0.077	0.825
TDCA	81.59 ± 87.85	0.81 ± 0.58	0.06 ± 0.07	0.020	0.003
Total conjugated secondary/tertiary BAs	189.91 ± 203.07	346.26 ± 305.59	111.69 ± 73.28	0.228	0.053

The data are the means ± SDs

^a^One-way ANOVA was used to compare differences between the GM vs. untreated groups and between the VCM vs. untreated groups

In this study, we first determined the BA composition of the colonic content to assess the influence of antibiotic-mediated microbial dysbiosis. The BA composition of the colonic content of GM-treated or VCM-treated mice was significantly altered when compared with that of untreated mice. In accordance with the microbial results, the proportion of conjugated primary BAs observed in GM-treated or VCM-treated mice was higher than that in untreated control animals and featured a 69-fold or 203-fold increase, respectively, in the proportion of detected TβMCA. Conversely, the proportions of unconjugated primary BAs and all unconjugated secondary Bas, including MCA, murocholic acid (MuroCA), DCA, and LCA, were lower in GM-treated or VCM-treated animals than in untreated animals (Fig. 3b and Table 2). We further explored alterations in the BA composition in the blood samples. The BA composition of the blood was markedly altered following GM or VCM treatment. The proportion of the FXR agonists CDCA and DCA decreased (10-fold and 30-fold, respectively) significantly in VCM-treated mice compared to that in untreated animals. GM treatment also significantly reduced the proportion of the FXR agonist DCA (55-fold). In contrast, the proportion of the FXR antagonist TβMCA increased five-fold and four-fold, respectively, after treatment with GM or VCM (Fig. 3b and Table 3). The hepatic BA composition reflects hepatic bile acid synthesis. The bile acid composition of the liver samples clearly differed between untreated animals and antibiotic-treated animals. In comparison to untreated mice, GM-treated mice exhibited a fourfold increase in the FXR agonist CDCA, occurring concomitant with a six-fold increase in the FXR antagonist TβMCA. Although VCM treatment increased the proportion of CDCA and TβMCA, the difference was not significant. The proportion of the FXR agonist DCA was similar in the untreated and treated animals (Fig. 3b and Table 4).

### Antibiotic treatment alters intestinal and hepatic FXR signaling in mice

**Fig. 4:**
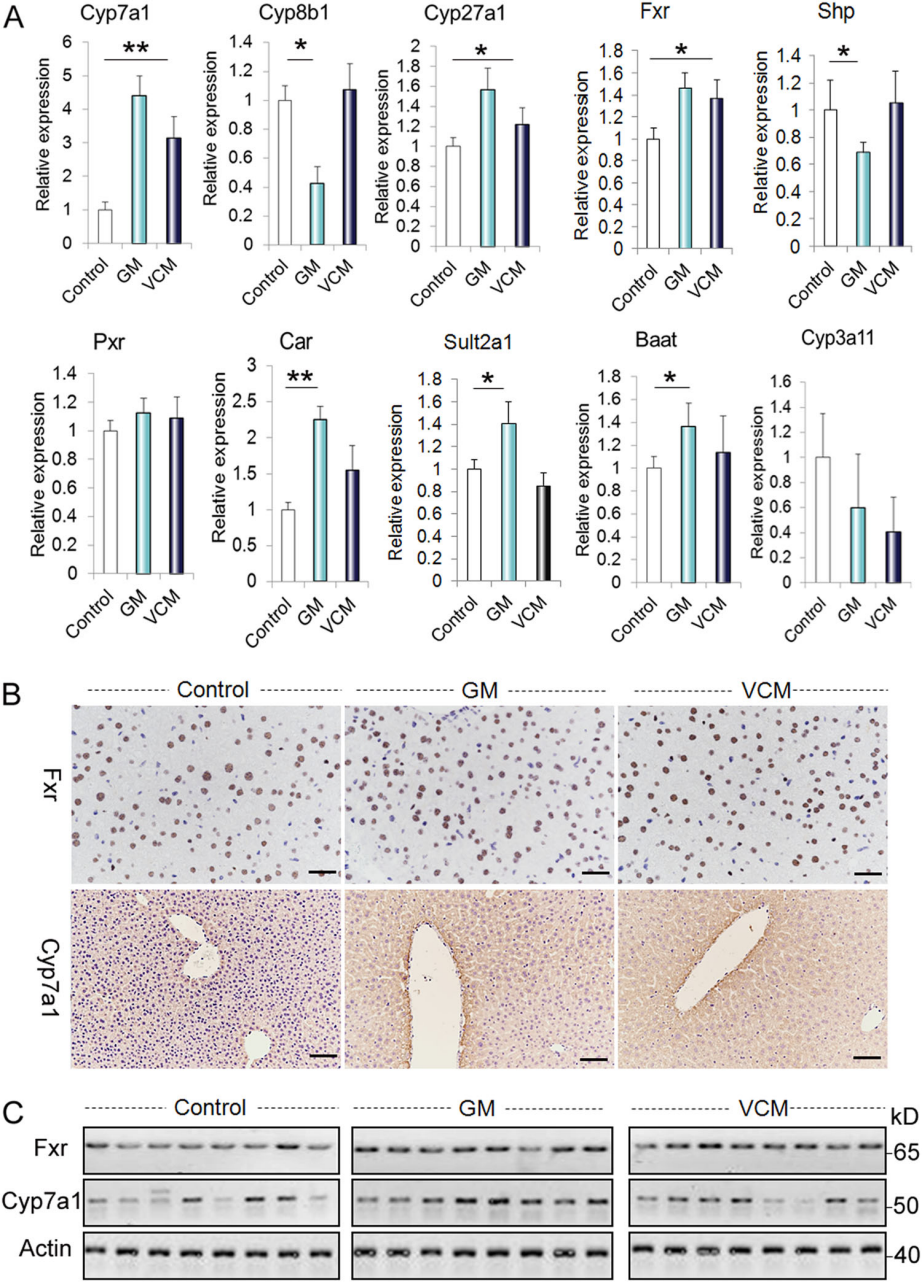
Analysis of the bile acid synthesis pathway in the liver. **a** Gene expression changes in bile acid synthesis enzymes and Fxr in untreated and antibiotic-treated animals. **b**, **c** Protein expression changes in the bile acid synthesis enzymes Cyp7a1 and Fxr in untreated and antibiotic-treated animals. Scale bar = 25 µm; *p < 0.05, **p < 0.01

**Fig. 5:**
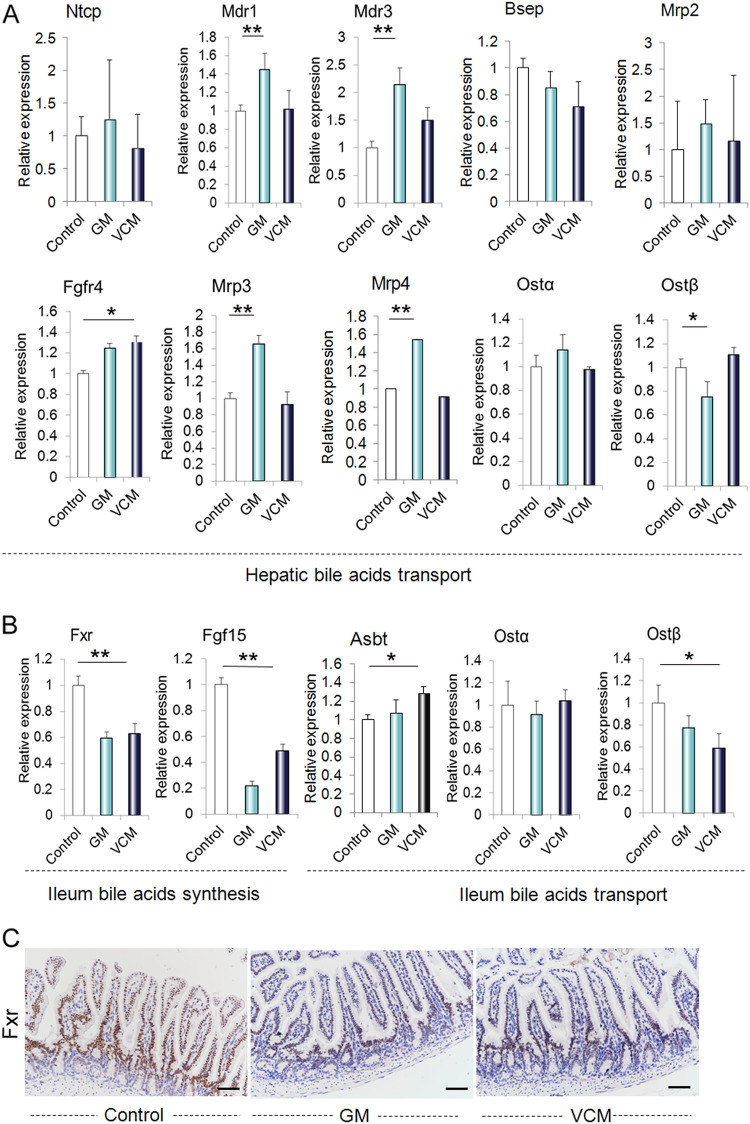
Analysis of bile acid synthesis and transport pathways in the liver and ileum. **a** Gene expression changes in bile acid transporters in the liver of untreated and antibiotic-treated animals. **b** Representative images of Fxr and Cyp7a1 immunohistochemical staining in liver tissues. **c** Western blot analysis for Fxr and Cyp7a1 in liver tissues. Scale bar = 25 µm; *p < 0.05, **p < 0.01

FXR is known to play a key role in the regulation of BA synthesis and homeostasis. FXR activation depends on the pattern of expressed isoforms and the BA pool composition, and we investigated the impact of antibiotic treatment on FXR activity by examining the gene and protein expression of key downstream FXR targets. To exclude the direct effect of antibiotics on liver and intestinal cells, we treated intestinal Caco2 cells and hepatic L02 cells with GM (2 g/L) or VCM (500 mg/L) for 16 h and found no evident changes in FXR expression (Supplementary Fig. 2). The ‘classical’ pathway producing the primary BAs CA and CDCA is initiated by CYP7A1, and the ‘alternative’ bile acid synthesis pathway is initiated by CYP27A1^20^. CYP8B1 directly converts CA into CDCA. In the rodent liver, the majority of CDCA is converted to α‑muricholic acid (α‑MCA) through the action of a 6β‑hydroxylase; α‑MCA can be further converted to β‑MCA by the epimerization of the 7α‑hydroxyl (OH) group of α-MCA to 7β‑OH^24^. As shown in Fig. 4, the relative expression of CYP7A1 mRNA as well as CYP7A1 protein and CYP27A1 mRNA was significantly higher in both GM-treated and VCM-treated animals than in untreated controls (Fig. 4a–c). GM decreased CYP8B1 gene expression, but there was no significant difference in CYP8B1 gene expression between control and VCM-treated animals (Fig. 4a). Following the conjugation of the primary bile acids CA, MCA, and CDCA to either taurine (predominantly in mice) or glycine (mainly in humans) by bile acyl-CoA synthetase (BACS) and bile acid-CoA:amino acid N-acyltransferase (BAAT) to form taurocholic acid (TCA), tauromuricholic acid (TMCA), taurochenodeoxycholic acid (TCDCA), glycocholic acid (GCA), and glycochenodeoxycholic acid (GCDCA), primary BAs are secreted from the liver into the bile canaliculus via the canalicular bile salt export pump (BSEP, also known as ABCB11)^25^. Concurrently, sulfated (catalyzed by enzymes such as sulfotransferase 2A1 (SULT2A1)) or glucuronidated (catalyzed by UDP-glucuronosyltransferase (UGT) enzymes) bile acids are amidated with either taurine or glycine and are secreted from the liver into the bile via multidrug resistance-associated protein 2 (MRP2, also known as ABCC2)^26^. The hepatic receptors CAR and PXR have a synergistic role in maintaining BA homeostasis in vivo, presumably through the combined induction of CYP3A4 and SULT2A1^27^. During cholestasis^28^, (when the flow of bile from the liver is slowed or blocked), excessive amounts of hepatic BAs and bilirubin can be excreted into the systemic circulation through basolateral export systems that are mediated by members of the MRP family, including MRP3 and MRP4, as well as sodium-independent organic anion-transporting polypeptide 2 (OATP2, also known as SLCO1B1) and the organic solute transporter subunit alpha (OSTα-OSTβ complex). We showed here that the relative mRNA expression of Car, Sult2a1, BAAT, and FXR-targeted genes such as Mdr1, Mrp3, and Mrp4 was higher in GM-treated but not VCM-treated animals than in untreated controls (Fig. 5a). Conversely, there was no significant difference in the expression of the FXR-targeted genes Bsep, Mrp2, and OSTα-OSTβ complex between the control and antibiotic-treated animals (Fig. 5a, b).

Following bile acid release into the intestinal lumen, both conjugated and unconjugated bile acids are reabsorbed in the distal ileum by the apical sodium-dependent bile acid transporter (ASBT, also known as SLC10A2), which is located in the brush border (that is, the apical microvillus membrane of small intestinal epithelial cells)^26^. These BAs are transported across enterocytes to the basolateral membrane, where the enteric OSTα-OSTβ complex facilitates transport into the portal vein^29^. Normal intestinal FXR activity maintains an efflux of BAs back into the portal vein and a controlled reuptake of BAs into enterocytes, which limits intracellular BA levels. Activation of intestinal FXRs upregulates the expression of fibroblast growth factor 15 (FGF15) in mice and that of its orthologue FGF19 in humans, which inhibit BA synthesis in hepatocytes via the activation of hepatic fibroblast growth factor receptor 4 (FGFR4)^30^. Activation of ileal FXRs by unconjugated bile acids downregulates the expression of ASBT and induces the expression of the OSTα-OSTβ complex. As shown in Fig. 5, the relative expression of FXR mRNA was significantly lower in GM- or VCM-treated animals than in untreated controls (Fig. 5b, c). Correspondingly, the FGF15 and OSTβ mRNA levels were significantly reduced in the GM-treated or VCM-treated animals (Fig. 5b).

## Discussion

Although previous studies have linked cholestasis to liver disease in patients with IF^3,31–34^, the underlying mechanisms are still not fully understood. In this study, we first describe a potential mechanism by which antibiotic administration contributes to the development of cholestasis in IF patients via the alteration of intestinal bacteria. We next show that following 2 weeks of oral gentamicin (GM) or vancomycin (VCM) treatment in mice, BA metabolism was significantly impaired, with a concomitant marked change in the intestinal microbiota composition. In contrast to VCM treatment, which mostly affected Gram-positive bacteria, GM mostly affected Gram-negative bacteria and was more important in maintaining BA homeostasis. In addition, FXR signaling contributed to VCM-induced and GM-induced BA dysmetabolism.

It is known that the gut microbiota plays an important role in BA biotransformation by controlling the deconjugation, dehydrogenation, dehydroxylation, and epimerization of primary bile acids in the distal small intestine and colon. Studies have demonstrated that members of *Lactobacilli*, *Bifidobacteria*, *Clostridium,* and *Bacteroides* deconjugate BAs by functional bile salt hydrolases (BSHs) in the human gut^16^. In the colon, it has been identified that bacteria of the genera *Clostridium* and *Eubacterium* can biotransform BAs into secondary bile acids by 7α-dehydroxylation^35^. Another major microbial bile acid biotransformation mechanism is epimerization, which alters the hydrophobicity and toxicity of BAs and protects the liver against highly toxic bile acids. *Bacteroides*, *Eubacterium*, *Clostridium*, *Escherichia*, *Eggerthella*, *Eubacterium,* and *Ruminococcus* produce enzymes that act on the 3-, 7-, and 12-position hydroxyl groups of bile acids to catalyze epimerization^36^. We showed that as expected, both GM and VCM treatment caused a reduction in the total bacterial abundance in the colonic contents. Interestingly, the abundances of the Gram-positive *Clostridium*, gram-positive *Bifidobacterium* and gram-negative *Eubacterium* genera were all reduced significantly following VCM (which mostly affected Gram-positive bacteria) or GM (which mostly affected Gram-negative bacteria) treatment. In accordance with the reduced abundance of biotransforming bacteria, the antibiotic-treated mice exhibited increased blood levels of primary BAs concurrent with decreased levels of secondary BAs.

FXR is a highly specific bile acid receptor that is activated by the hydrophobic bile acids CDCA, DCA, and LCA at physiological concentrations in order to directly stimulate the transcription of genes that mediate the synthesis, transport, and absorption of bile acids and reverse cholesterol transport. It has been shown that FXR activation is strongly influenced by BA composition; the activating potency of specific BA species is chenodeoxycholic acid (CDCA) > deoxycholic acid (DCA) > lithocholic acid (LCA) > cholic acid (CA)^37^. In mice, it has been reported that taurine-conjugated β-muricholic acid (TβMCA) is the FXR antagonist. It has been reported that the presence of a gut microbiota that reduces the levels of TβMCA resulted in the increased expression of FGF15 through FXR activation^15^. Here, we showed that both GM and VCM administration could increase the levels of TβMCA in the colonic contents. Consistently, ileal FXR activation was inhibited and further resulted in a decrease in FGF15 production. In the liver, we observed a failure of the FXR-FGF15 pathway to appropriately increase BA synthesis by promoting Cyp7a1 and Cyp27a1 gene expression. Given that Kim et al.^38^ identified the FXR antagonist TβMCA, which cannot be metabolized in the absence of bacteria, it may be concluded that GM or VCM treatment decreased the abundance of the intestinal microbiota and increased BA synthesis via the downregulation of the FXR-FGF15 feedback mechanism. In this study, we also showed that antibiotic treatment altered BA transport in the liver and ileum through the FXR. In the liver, antibiotic treatment, especially GM treatment, increased the expression of FXR target genes, including Mrp3, Mrp4, Mdr1, and Mdr3, that promote the symmetric circulation of BA. In the ileum, GM or VCM treatment reduced the expression of the FXR target genes Ostα/Ostβ and resulted in decreased BA uptake in the ileum. This effect is consistent with the hypothesis that FXR maintains BA homeostasis by mediating communication between the intestine and the liver.

**Fig. 6:**
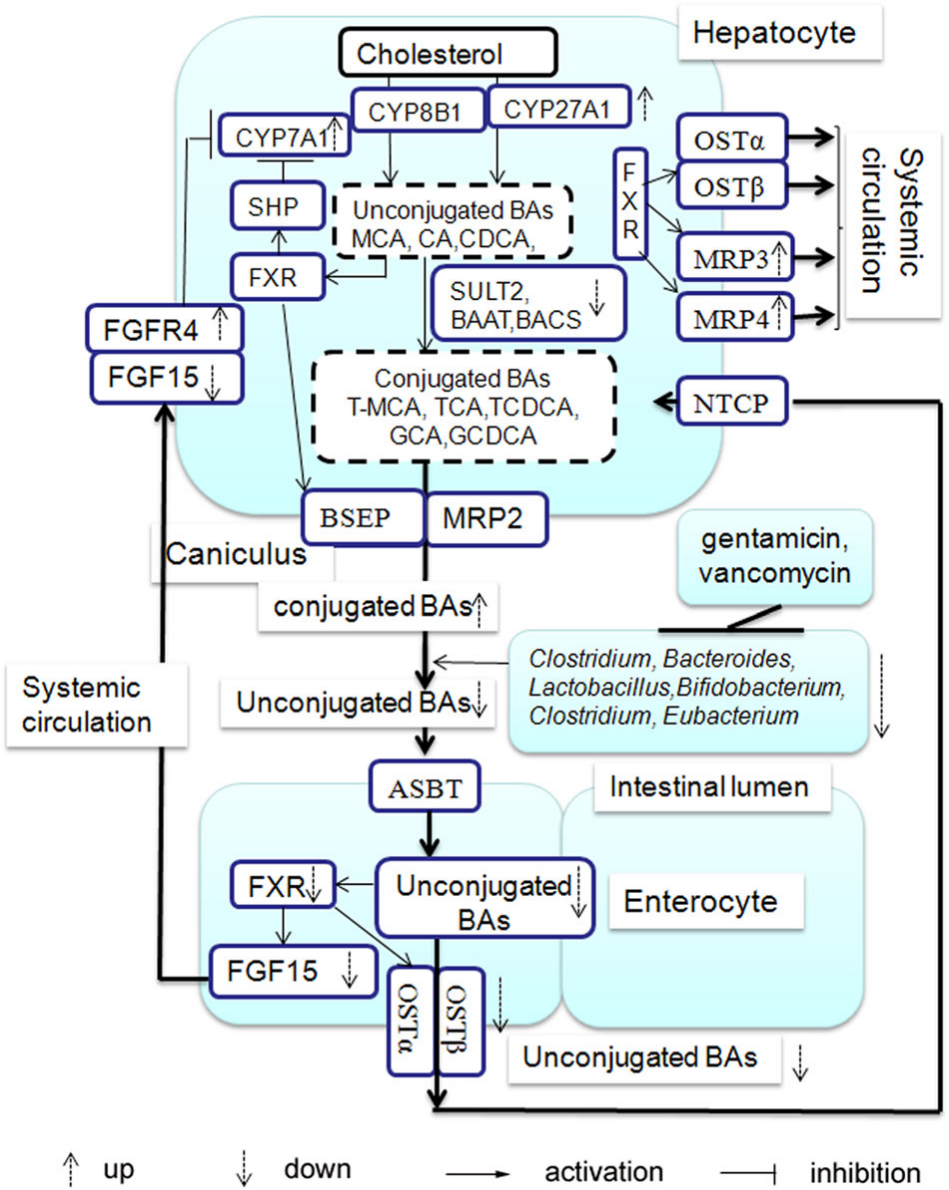
Schematic depicting the potential influence of antibiotic treatment on bile acid dysmetabolism Antibiotic (gentamicin, which mainly affects Gram-negative bacteria or vancomycin, which mainly inhibits Gram-positive bacteria) administration reduced or depleted the abundance of bile acid (BA)-biotransforming bacteria. The main bacterial genera of the gut bacteria involved in BA metabolism included *Bacteroides*, *Clostridium, Lactobacillus, Eubacterium*, and *Bifidobacterium*, which mediate BA deconjugation and oxidation; the epimerization of hydroxyl groups at C3, C7 and C12; 7-dehydroxylation; and desulfation. The depletion of BA-biotransforming bacteria caused BA dysmetabolism with an alteration of the BA composition, reflected by the increased proportion of conjugated primary BAs and the decreased proportion of unconjugated BAs. In the terminal ileum, the low levels of unconjugated BA (including FXR agonist CDCA) could not activate the FXR and correspondingly reduced the production of FGF19/15, which resulted in increased BA synthesis in the liver by upregulating the enzymes CYP7A1, CYP8B1, and CYP27A1

In summary, we have demonstrated that antibiotics have a profound systemic effect on BA metabolism, which augments the understanding of the mechanisms that underlie the development of liver disease in pediatric patients with IF. Not only do antibiotics exert their effects within the gut, but they also affect other parts of the enterohepatic system, such as by regulating BA synthesis in the liver (Fig. 6).

## Electronic supplementary material

Supplemental data
