# Correction: Administration of antibiotics contributes to cholestasis in pediatric patients with intestinal failure via the alteration of FXR signaling

**DOI:** 10.1038/s12276-022-00852-7

**Published:** 2022-09-28

**Authors:** Yongtao Xiao, Kejun Zhou, Ying Lu, Weihui Yan, Wei Cai, Ying Wang

**Affiliations:** 1 Department of Pediatric Surgery, Xin Hua Hospital, School of Medicine, Shanghai Jiao Tong University, Shanghai, China; 2 Shanghai Institute of Pediatric Research, Shanghai, China; 3 Shanghai Key Laboratory of Pediatric Gastroenterology and Nutrition, Shanghai, China

Correction to: *Experimental & Molecular Medicine* 10.1038/s12276-018-0181-3, published online 30 November 2018

After online publication of this article, the authors noticed an error in the Table 3.

The correct statement of this article should have read as below.

**Table 3:** Bile acid profiles in the serum of mice (nmol/L).

Bile acid (nM)	Untreated	GM	VCM	*p* value^a^ (GM vs. untreated)	*p* value^a^ (VCM vs. untreated)
CDCA	50.18 ± 56.02	39.10 ± 36.22	4.84 ± 9.10	0.625	0.040
ωMCA	426.83 ± 466.26	346.65 ± 296.62	9.90 ± 18.38	0.669	0.034
αMCA	61.03 ± 73.34	44.95 ± 40.58	9.63 ± 14.24	0.573	0.202
βMCA	389.17 ± 320.73	1444.09 ± 1248.86	158.44 ± 260.10	0.026	0.127
HCA	7.91 ± 7.84	7.12 ± 5.51	0.67 ± 0.76	0.807	0.099
CA	308.85 ± 313.95	612.60 ± 602.89	85.76 ± 223.06	0.199	0.116
muroCA	13.79 ± 14.79	2.03 ± 1.71	0.74 ± 0.56	0.078	0.113
12DHCA	19.89 ± 22.39	21.28 ± 12.15	2.34 ± 3.37	0.877	0.061
3DHCA	7.39 ± 8.45	14.24 ± 17.99	0.86 ± 1.77	0.317	0.119
7DHCA	229.47 ± 266.30	296.54 ± 282.54	3.51 ± 3.48	0.611	0.087
Total unconjugated primary BA	1514.51 ± 1434.41	2825.56 ± 2413.35	268.00 ± 525.23	0.180	0.035
TωMCA	408.15 ± 333.22	436.86 ± 89.64	188.22 ± 141.25	0.806	0.126
TαMCA	155.97 ± 84.02	233.22 ± 135.63	139.45 ± 238.28	0.166	0.848
TβMCA	802.86 ± 812.95	4165.92 ± 2223.07	3379.12 ± 2887.32	0.001	0.021
THCA	2.76 ± 2.85	3.93 ± 3.37	3.05 ± 1.53	0.497	0.899
TCA	279.05 ± 156.02	867.00 ± 707.31	901.54 ± 978.74	0.027	0.078
TCDCA	25.54 ± 15.85	60.19 ± 69.22	161.05 ± 145.27	0.163	0.014
GCDCA	1.97 ± 0.12	2.02 ± 0.11	1.94 ± 0.14	0.451	0.735
GCA	3.07 ± 2.23	2.96 ± 0.78	1.77 ± 0.31	0.889	0.185
Total conjugated primary BA	1678.01 ± 907.35	5771.19 ± 3069.06	4749.39 ± 4189.80	0.001	0.048
6ketoLCA	3.28 ± 1.83	1.87 ± 1.09	0.96 ± 0.40	0.116	0.075
7ketoLCA	12.12 ± 12.96	4.74 ± 3.09	0.74 ± 0.94	0.119	0.185
UDCA	123.85 ± 140.70	193.94 ± 163.05	15.40 ± 27.41	0.343	0.119
DCA	327.57 ± 228.46	5.91 ± 5.45	11.32 ± 23.28	0.006	0.006
Total unconjugated secondary/tertiary BA	388.89 ± 391.37	204.08 ± 165.04	18.75 ± 27.34	0.210	0.018
HDCA	47.92 ± 40.00	3.03 ± 2.06	0.05 ± 0.06	0.007	
TLCA	3.54 ± 4.70	0.72 ± 0.80	1.58 ± 2.42	0.145	0.346
TUDCA	83.14 ± 61.87	253.81 ± 140.48	350.96 ± 245.35	0.004	0.007
THDCA	67.38 ± 28.25	2.16 ± 1.18	278.43 ± 241.41	0.005	0.038
TDCA	172.61 ± 107.01	1.80 ± 1.19	2.04 ± 1.72	0.011	0.026
Total conjugated secondary/tertiary BA	329.00 ± 160.17	258.59 ± 139.70	369.78 ± 213.00	0.335	0.659

The data are the means ± SDs.

^a^One-way ANOVA was used to compare differences between the GM vs. untreated groups and between the VCM vs. untreated groups.

“The authors unintentionally uploaded Table 2 as Table 3 when submitting revised manuscript. The authors corrected this error using the right Table 3.”

The authors apologize for any inconvenience caused.

The original article has been corrected.

