# Supplementary material for: Administration of antibiotics contributes to cholestasis in pediatric patients with intestinal failure via the alteration of FXR signaling
Source: Exp Mol Med. 2018 Nov 30;50(12):1–14. doi: 10.1038/s12276-018-0181-3 (PMC6269533; doi:10.1038/s12276-018-0181-3)
Supplement: Supplementary file 1 — Supplemental data [file 12276_2018_181_MOESM1_ESM.docx]

**Supplemental data**

**Administration of antibiotics contributes to cholestasis in pediatric patients with intestinal failure via the alteration of FXR signaling**

Yongtao Xiao, Kejun Zhou, Ying Lu, Weihui Yan, Wei Cai and Ying Wang

**Supplementary Methods**

**Supplementary Table 1-3**

**Supplementary Figure 1,2**

**Supplementary Methods**

**Biochemical measurements and Enzyme Linked Immunosorbent Assay (ELISA)**

The human blood samples were analyzed for alanine aminotransferase (ALT), aspartate aminotransferase (AST), alkaline phosphatase (ALP) and bilirubin and conjugated bilirubin by using routine hospital laboratory methods.. For ELISA analysis, the human IL-6 Platinum ELISA (BMS213, eBioscience) Kit, human TNF-alpha Platinum ELISA (BMS223, eBioscience) Kit and human FGF19 ELISA Kit (R&D Systems, MN,USA) were used in this study according to protocols of manufactures.

**Bile acids composition measurements**

Bile acids measurements were performed with a Waters ACQUITY ultra performance liquid chromatography coupled with Waters Xevo TQ-S triple quadrupole mass spectrometry according to the previously reported method [^1^](#_ENREF_1)^,^[^2^](#_ENREF_2). Data acquisition and bile acids quantification were performed using the MassLynx 4.1 software (Waters). The bile acid inculded the cholic acid (CA), glycocholic acid (GCA), taurocholic acid (TCA), chenodeoxycholic acid (CDCA), glycochenodeoxycholic acid (GCDCA), taurochenodeoxycholic acid (TCDCA), deoxycholic acid (DCA), glycodeoxycholic acid (GDCA), taurodeoxycholic acid (TDCA), ursodeoxycholic acid (UDCA), glycoursodeoxycholic acid (GUDCA), tauroursodeoxycholic acid (TUDCA), lithocholic acid (LCA), glycolithocholic acid (GLCA), taurolithocholic acid ( TLCA), hyocholic acid (HCA), glycohyocholic acid (GHCA), taurohyocholic acid (THCA), α-muricholic acid (αMCA), tauro-α-muricholic acid (TαMCA), β-muricholic acid (βMCA), tauro- β-muricholic acid ( TβMCA), ω-muricholic acid (ωMCA), tauro-ω-muricholic acid (TωMCA), hyodeoxycholic acid (HDCA), glycohyodeoxycholic acid (GHDCA), taurohyodeoxycholi acid (THDCA), murocholic acid (MuroCA), dehydrocholic acid (DHCA), glycodehydrocholic acid (GDHCA), taurodehydrocholic acid (TDHCA), 3-dehydrocholic acid (3-DHCA), 7-dehydrocholic acid (7-DHCA), isodeoxycholic acid (isoDCA), apocholic acid (apoCA), 6-ketolithocholic acid (6-KLCA), 7-ketolithocholic acid (7-KLCA), 12-ketolithocholic acid (12-KLCA), 23-nordeoxycholic acid (23norDCA), dehydrolithocholic acid (DHLCA). Deuterated internal standards (IS) lithocholic acid-2,2,4,4-D4 (LCA-D4), cholic acid-2,2,4,4-D4 (CA-D4) and Chenodeoxycholic Acid 24-Acyl-β-D-glucuronide (CDCA-24G), and Sodium Taurochlate -2, 2, 4, 4-D4(TCA-D4).

**Quantitative real-time polymerase chain reaction (qRT-PCR)**

The liver and intestinal samples from mice were homogenized using MagNA Lyser Instrument and MagNA Lyser Green Beads (Manassas, VA, USA). Total RNA was extracted with Trizol according to the protocol of the manufacture (Invitrogen, Foster, CA). cDNA synthesized from 1 μg of total RNA with a High Capacity cDNA Reverse Transcription kit (Applied Biosystems, Foster City, CA). A SYBR-Green Universal Master Mix kit (Applied Biosystems, Foster City, CA) was employed to detect the levels of the genes. The primers are listed in bellowed: The Cyp7a1: 5’-CCTCTGGGCATCTCAAGCAA-3’ and 5’-AATGGC-

ATTCCCTCCAGAGC-3’; Cyp8b1: 5’- TTGCAAATGCTGCCTCAACC-3’ and 5’- TAACAGTCGCACACATGGCT-3’; Cyp27a1: 5’- GAGTACGGAGGGTCC-

AGGAA-3’ and 5’- GTCCCAAAGGAGGTTGTCCA -3’; Baat: 5’-CATCTGTGCTGACCGACAGG-3’ and 5’- ACAAGAGCACTGAGAGGAA-

CA-3’; CYP3A11: 5’- CCTGGGTGCTCCTAGCAATC -3’ and 5’- GGCCCAGGAATTCCCTGTTT -3’; Sult2a1: 5’- GCTGGATCTCGTCCTCAA

-GT-3’ and 5’- GAAGGCTTCAGCTTGGGCTA-3’; Slc10a1 (Ntcp) 5’- CCCCCTGAAGTCATTGGACC--3’ and 5’- ATCTTCTGTTGCAGCAGCCT -3’; ABCB11 (Bsep) 5’-ACATCTGTAGGGTTGTTGAGTGA-3’ and 5’- CAAAGAAGCCAACTCGAGCG-3’; ABCC2 (Mrp2): 5’- ACATCTGCTTCCCTTGAGGC-3’ and 5’- TCGGCTTCCGGACTTTTCAA-3’; ABCC4 (Mrp4): 5’-CACACCGAGGTGAAACCCAA-3’ and 5’- CCTCTCCGAGGTGCTTTGAG-3’; OSTα: 5’- CAGCGTCTGCCTGAGAGAAA-3’ and 5’-GGTGAGGGCTATGTCCACTG-3’. OSTβ: 5’- GAAACATGGACCACAGTGCAG-3’ and 5’- GCCAGGACCAGGATGGAATAA-3’; Slc10a2 (Asbt): 5’- CTTCTCCCCCGAGGATCTCA-3’ and 5’-TGATGGCCTGGAGTCCATTTC-3’; GpBAR1 (Tgr5): 5’-CTTCTCTCTGTCCGCGTGTT-3’ and 5’- GCCAGGGTTGAGGGTACATC-3’ Fgf15: 5’- ACGGCAAGATATACGGGCTG-3’ and 5’- GGCTTGGCCTGGATGAAGAT-3’. Fgfr4: 5’- ATTCCTGGCTCTTCGGCCC-3’ and 5’- CAGACTTCCCACTGACCACC-3’. Mrp3: 5’- TACAGGAAGGCTCTGGTCAT-3’ and 5’-GGATCTGCCAGAGGAAGTAT-3’. Mdr3: 5’- GACATGTC-

TTCAAAAGATTCTGGAT-3’ and 5’-TCCAGGGCCTCTTTGGTACT-3’. Fxr-5’-GGGATGTTGGCTGAATGTATGT-3’ and 5’-CAGCGTGCTGCTTCACATTT-3’. SHP: 5’- AGGGCACGATCCTCTTCAAC-3’ and 5’- AGGGCTCCAAGACTTCACAC-3’. Pxr: 5’- GGGTTCCAATGAAGATCTCTCTGC-3’ and 5’-CTGGTCCTCAATAGGCAGGTC-3’. Car: 5’- GGAAGGTGTGAGGTCAGCAA-3’ and 5’- GGACCAGTTCTTTCTGCTGC-3’ Actin: 5’-GCAGATGTGG-

ATCAG-CAAGC-3’ and 5’- AGGGTGTAAAACGCAGCTCAG-3’.

**Supplementary Table 1-3**

**Supplementary Talbe 1. The bile acid composition in feces (nmol/mg)**

|  | **Without Cholestasis**  **(n=18)** | | | **With Cholestasis**  **(n=16)** | | | **p value *** |
| --- | --- | --- | --- | --- | --- | --- | --- |
| **CA** | 348.412 | ± | 174.999 | 457.326 | ± | 126.643 | 0.046 |
| **CDCA** | 219.634 | ± | 144.065 | 306.390 | ± | 137.112 | 0.089 |
| **HCA** | 20.949 | ± | 20.686 | 13.837 | ± | 23.809 | 0.377 |
| **GCA** | 28.454 | ± | 48.861 | 35.309 | ± | 43.220 | 0.673 |
| **TCA** | 74.374 | ± | 144.506 | 91.631 | ± | 217.836 | 0.799 |
| **GCDCA** | 19.951 | ± | 27.531 | 26.616 | ± | 45.204 | 0.629 |
| **TCDCA** | 42.346 | ± | 87.321 | 60.278 | ± | 128.763 | 0.656 |
| **GHCA** | 0.500 | ± | 0.938 | 0.409 | ± | 0.963 | 0.788 |
| **THCA** | 2.187 | ± | 8.051 | 1.507 | ± | 4.992 | 0.767 |
| **LCA** | 6.589 | ± | 20.296 | 4.102 | ± | 0.138 | 0.171 |
| **HDCA** | 0.217 | ± | 0.353 | 0.091 | ± | 0.086 | 0.144 |
| **DCA** | 43.200 | ± | 96.894 | 8.816 | ± | 2.392 | 0.064 |
| **UDCA** | 46.408 | ± | 77.538 | 42.049 | ± | 112.960 | 0.902 |
| **GLCA** | 0.205 | ± | 0.735 | 0.011 | ± | 0.012 | 0.257 |
| **GDCA** | 1.912 | ± | 4.465 | 0.026 | ± | 0.047 | 0.074 |
| **GUDCA** | 5.234 | ± | 15.848 | 1.040 | ± | 3.476 | 0.270 |
| **TUDCA** | 13.992 | ± | 51.944 | 4.357 | ± | 13.987 | 0.444 |
| **Total** | 874.564 | ± | 515.403 | 1041.794 | ± | 499.638 | 0.355 |

Data are means ± SD

*Comparison between patients with and without cholestasis using Fisher’s exact test or Mann Whitney U-test

**Supplementary Talbe 2. The bile acid composition in serum (nmol/L)**

|  | **Without Cholestasis** | | | **With Cholestasis** | | | **p value*** |
| --- | --- | --- | --- | --- | --- | --- | --- |
| **CA** | 72.106 | ± | 121.566 | 1725.809 | ± | 2928.253 | 0.031 |
| **CDCA** | 97.313 | ± | 205.879 | 875.123 | ± | 1299.198 | 0.024 |
| **HCA** | 4.782 | ± | 5.016 | 43.855 | ± | 62.539 | 0.049 |
| **GCA** | 446.519 | ± | 581.230 | 744.536 | ± | 1146.184 | 0.347 |
| **TCA** | 322.888 | ± | 614.917 | 787.391 | ± | 1776.895 | 0.324 |
| **THCA** | 19.206 | ± | 27.264 | 238.777 | ± | 989.074 | 0.382 |
| **GHCA** | 63.847 | ± | 85.431 | 214.375 | ± | 622.962 | 0.361 |
| **GCDCA** | 851.969 | ± | 863.286 | 1182.582 | ± | 1593.940 | 0.457 |
| **TCDCA** | 376.613 | ± | 382.709 | 858.868 | ± | 2002.389 | 0.350 |
| **LCA** | 3.243 | ± | 1.240 | 1.842 | ± | 3.414 | 0.362 |
| **DCA** | 17.963 | ± | 20.810 | 13.168 | ± | 15.730 | 0.441 |
| **UDCA** | 48.457 | ± | 66.166 | 224.110 | ± | 528.390 | 0.395 |
| **TLCA** | 1.911 | ± | 4.874 | 4.550 | ± | 13.780 | 0.587 |
| **GDCA** | 13.623 | ± | 30.706 | 158.238 | ± | 348.575 | 0.149 |
| **TDCA** | 19.267 | ± | 16.344 | 360.133 | ± | 379.806 | 0.050 |
| **TUDCA** | 6.513 | ± | 8.667 | 244.454 | ± | 684.640 | 0.343 |
| **GUDCA** | 105.336 | ± | 117.367 | 658.535 | ± | 1330.462 | 0.183 |
| **Total** | 2385.05 | ± | 2040.519 | 7687.304 | ± | 8483.365 | 0.0197 |

Data are means ± SD

*Comparison between patients with and without cholestasis using Fisher’s exact test or Mann Whitney U-test

**Supplementary Talbe 3. The bile acid composition in liver (nmol/mg)**

|  | **Without Cholestasis** | | | **With Cholestasis** | | | **p value*** |
| --- | --- | --- | --- | --- | --- | --- | --- |
| **CA** | 0.377 | ± | 0.222 | 3.588 | ± | 4.525 | 0.011 |
| **CDCA** | 0.061 | ± | 0.087 | 0.487 | ± | 0.760 | 0.040 |
| **GCA** | 115.630 | ± | 74.624 | 103.916 | ± | 50.831 | 0.628 |
| **TCA** | 336.786 | ± | 341.759 | 86.059 | ± | 77.512 | 0.012 |
| **TCDCA** | 176.169 | ± | 165.029 | 59.231 | ± | 86.377 | 0.026 |
| **GCDCA** | 158.908 | ± | 122.276 | 127.354 | ± | 104.071 | 0.462 |
| **DCA** | 0.614 | ± | 0.328 | 0.549 | ± | 0.315 | 0.589 |
| **UDCA** | 0.028 | ± | 0.032 | 0.081 | ± | 0.149 | 0.248 |
| **TUDCA** | 4.385 | ± | 8.172 | 0.477 | ± | 0.668 | 0.113 |
| **GUDCA** | 3.744 | ± | 7.104 | 7.352 | ± | 9.813 | 0.264 |
| **Total** | 795.822 | ± | 647.491 | 389.0135 | ± | 277.0113 | 0.038 |

Data are means ± SD

*Comparison between patients with and without cholestasis using Fisher’s exact test or Mann Whitney U-test


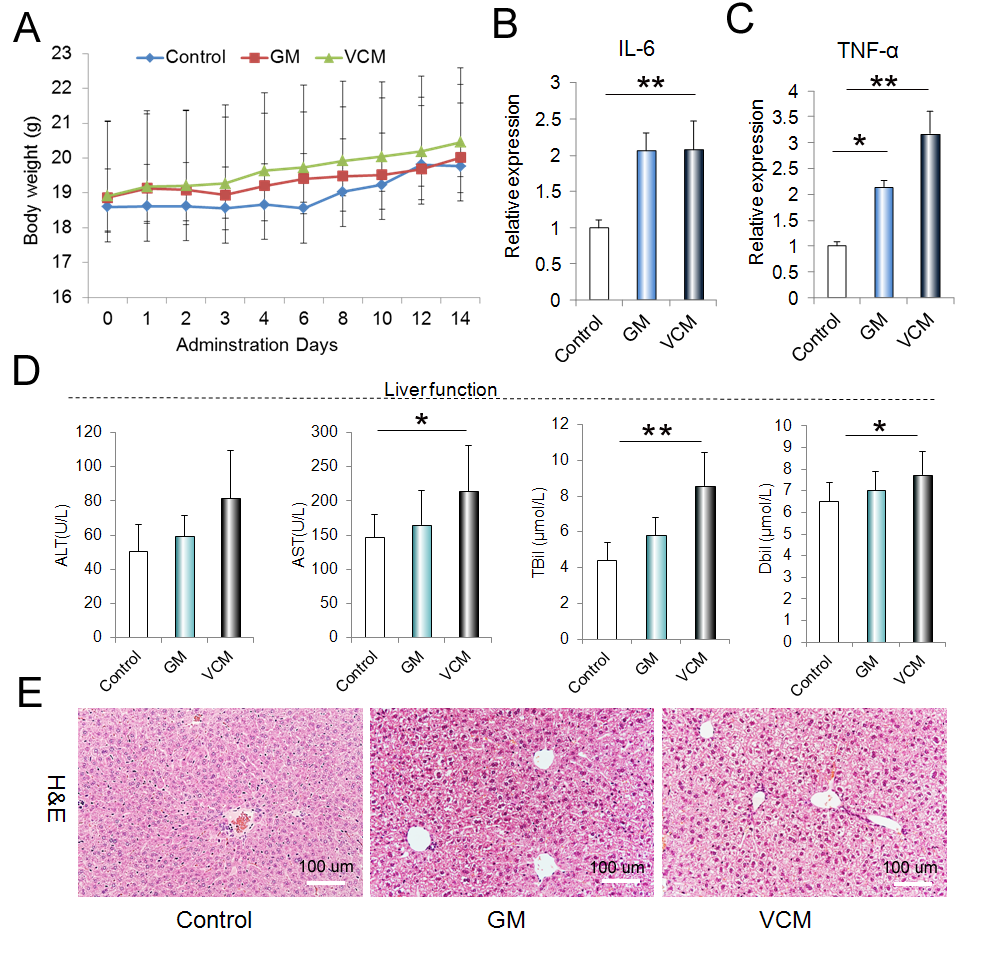


**Supplementary Figure 1: The changes of liver function and inflammation in mice treated with GM or VCM.** (A) The body weight changes of GM group (n=10), VCM group (n=10) and untreated mice (n=12). (B, C) Gene expression of IL-6 and TNF-α in liver. (D) The liver fucntion makers including Alanine Transaminase (ALT), AST Aspartate Transaminase (AST), total bilirubin (TBil) and direct bilirubin (TBil) altered in the presence of GM or VCM. (E) Histological changes in the liver. *p <0.05, ** p <0.01


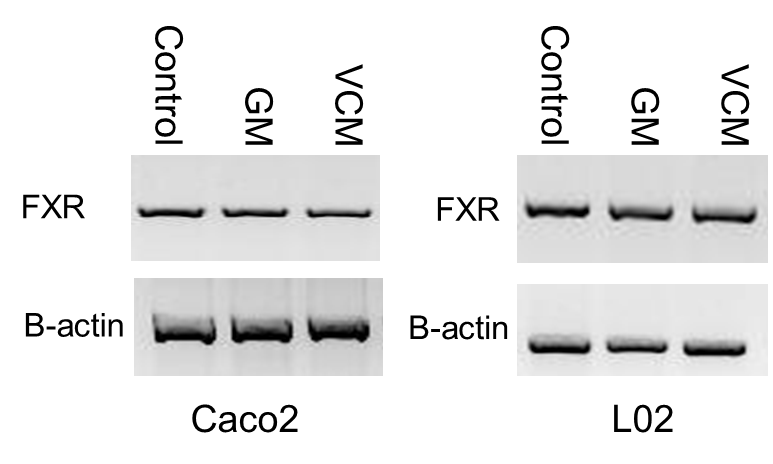


**Supplementary Figure 2: The effects of VCM or GM treaments on the FXR expression in Caco2 and L02 cells.** The intestinal Caco2 cells and L02 hepatic cells were treated with GM (2 g/L) or VCM (500 mg/L) for 16 hours, and the changes of FXR expression were detected by Western-blot.

**Supplementary References**

1. Xie, G. *et al.* Alteration of bile acid metabolism in the rat induced by chronic ethanol consumption. *FASEB J* **27**, 3583-93 (2013).

2. Garcia-Canaveras, J.C., Donato, M.T., Castell, J.V. & Lahoz, A. Targeted profiling of circulating and hepatic bile acids in human, mouse, and rat using a UPLC-MRM-MS-validated method. *J Lipid Res* **53**, 2231-41 (2012).
